# Growth Process and CQDs-modified Bi_4_Ti_3_O_12_ Square Plates with Enhanced Photocatalytic Performance

**DOI:** 10.3390/mi10010066

**Published:** 2019-01-18

**Authors:** Xinxin Zhao, Hua Yang, Ziming Cui, Xiangxian Wang, Zao Yi

**Affiliations:** 1 State Key Laboratory of Advanced Processing and Recycling of Non-ferrous Metals, Lanzhou University of Technology, Lanzhou 730050, China; zhaoxinxin7520@163.com (X.Z.); cuizim1983@163.com (Z.C.); wangxx869@126.com (X.W.); 2 Joint Laboratory for Extreme Conditions Matter Properties, Southwest University of Science and Technology, Mianyang 621010, China; yizaomy@swust.edu.cn

**Keywords:** Bi_4_Ti_3_O_12_ square plates, CQDs, CQDs@Bi_4_Ti_3_O_12_ composites, photodegradation of RhB, photocatalytic mechanism

## Abstract

Bi_4_Ti_3_O_12_ square plates were synthesized via a hydrothermal route, and their growth process was systematically investigated. Carbon quantum dots (CQDs) were prepared using glucose as the carbon source, which were then assembled on the surface of Bi_4_Ti_3_O_12_ square plates via a hydrothermal route with the aim of enhancing the photocatalytic performance. XRD (X-ray powder diffraction), SEM (scanning electron microscopy), TEM (transmission electron microscopy), UV-vis DRS (diffuse reflectance spectroscopy), XPS (X-ray photoelectron spectroscopy), FTIR (Fourier transform infrared spectroscopy), PL (photoluminescence) spectroscopy, EIS (electrochemical impedance spectroscopy) and photocurrent spectroscopy were used to systematically characterize the as-prepared samples. It is demonstrated that the decoration of CQDs on Bi_4_Ti_3_O_12_ plates leads to an increased visible light absorption, slightly increased bandgap, increased photocurrent density, decreased charge-transfer resistance, and decreased PL intensity. Simulated sunlight and visible light were separately used as a light source to evaluate the photocatalytic activity of the samples toward the degradation of RhB in aqueous solution. Under both simulated sunlight and visible light irradiation, CQDs@Bi_4_Ti_3_O_12_ composites with an appropriate amount of CQDs exhibit obviously enhanced photocatalytic performance. However, the decoration of excessive CQDs gives rise to a decrease in the photocatalytic activity. The enhanced photocatalytic activity of CQDs-modified Bi_4_Ti_3_O_12_ can be attributed to the following reasons: (1) The electron transfer between Bi_4_Ti_3_O_12_ and CQDs promotes an efficient separation of photogenerated electron/hole pairs in Bi_4_Ti_3_O_12_; (2) the up-conversion photoluminescence emitted from CQDs could induce the generation of additional electron/hole pairs in Bi_4_Ti_3_O_12_; and (3) the photoexcited electrons in CQDs could participate in the photocatalytic reactions.

## 1. Introduction

To date, water pollution particularly caused by organic dyes and pigments generated from chemical industries is getting increasingly serious and poses a great threat to aquatic life and human health. In the context of energy shortage, how to use solar energy as the power source to deal with water pollution has become an important subject. To achieve this aim, recently, semiconductor-based photocatalysis has aroused tremendous interest [1,2,3,4,5,6]. Titanium dioxide and titanium-based oxide semiconductors have been extensively studied as an important class of semiconductor photocatalysts. Nevertheless, these semiconductor photocatalysts generally have a wide bandgap (*E*_g_) greater than 3.0 eV and are photocatalytically active only under ultraviolet (UV) irradiation. This limits the utilization rate of solar energy. Furthermore, the photoexcited electrons (e^−^) and holes (h^+^) in the semiconductors easily undergo rapid geminate recombination, and thus, only a small fraction of the photoexcited carriers are able to participate in the photocatalytic reactions. This implies that the photon utilization rate is very low. Widening the light absorption range and promoting the photoexcited electron/hole pair separation are two of the key points to achieve an excellent photocatalyst with the best use of solar energy. Therefore, many strategies have been developed to modify semiconductor photocatalysts [7,8,9,10,11,12,13,14,15,16].

Low-dimension nanomaterials like graphene, carbon quantum dots (CQDs) and SiC nanowires offer great potential applications for biological labeling, bioimaging, drug delivery, energy conversion, optoelectronic devices, wave absorption and sensors due to their attractive properties [17,18,19,20,21,22,23,24,25,26,27,28]. In particular, photoexcited CQDs are excellent electron donors and electron acceptors. The interesting photoinduced electron transfer property suggests that CQDs can be used to modify photocatalysts with the aim of promoting the separation of photogenerated electron/hole pairs. Moreover, the PL up-conversion effect allows CQDs to harvest long-wavelength light (visible/near-infrared (vis/NIR) light) and then emit short-wavelength light (UV light). The emitted UV light can stimulate the generation of electron/hole pairs in wide-bandgap semiconductors like titanium-based oxides. Assembly of CQDs on the surface of semiconductors has become an important strategy for the design of excellent semiconductor photocatalysts capable of making full use of solar energy. Several examples of CQDs-modified photocatalysts with enhanced photocatalytic performance include CQDs/Bi_2_WO_6_, α-Bi_2_O_3_/C-dots, CQDs/BiOBr, and CQDs/g-C_3_N_4_ [29,30,31,32].

As an important titanium-based oxide semiconductor, bismuth titanate (Bi_4_Ti_3_O_12_) has been extensively studied owing to its pronounced photocatalytic activity toward the degradation of organic pollutants and water splitting into hydrogen [33,34,35,36,37]. Bi_4_Ti_3_O_12_ is composed of perovskite-like (Bi_2_Ti_3_O_10_)^2^^−^ blocks regularly interleaved with (Bi_2_O_2_)^2+^ units, forming a special layer structure along the [010] direction (b-axis) [38]. A polarization electric field is formed perpendicular to the (010) facet. Under the action of the polarization electric field, photogenerated electrons and holes are easily separated and migrate along the [010] direction to reach the (010) facet [39]. The anisotropic layered structure suggests that the photocatalytic activity of Bi_4_Ti_3_O_12_ highly depends on its morphology. In our previous work, we synthesized large-sized Bi_4_Ti_3_O_12_ nanosheets with a highly exposed (010) facet via a hydrothermal route and found that their photocatalytic activity was much higher than that of Bi_4_Ti_3_O_12_ nanoparticles [33]. On the other hand, Bi_4_Ti_3_O_12_ was frequently integrated with other semiconductors or decorated with noble metals to achieve excellent composite photocatalysts [40,41,42,43,44,45,46,47,48]. Very recently, Wang et al. reported on bamboo prepared CQDs for enhancing Bi_4_Ti_3_O_12_ photocatalytic activity [49]. Their work demonstrated that CQDs were a good modifier to enhance the photocatalytic activity of Bi_4_Ti_3_O_12_, although only 65% of ciprofloxacin was observed to be degraded at 90 min of photocatalysis. Further increasing the photocatalytic performance and elucidating the enhanced photocatalytic mechanism of CQDs-modified Bi_4_Ti_3_O_12_ is still necessary. Here we report on an alternative method to assemble CQDs on Bi_4_Ti_3_O_12_ square plates. In the present study, glucose was used as the carbon source to produce CQDs. The preparation of CQDs and Bi_4_Ti_3_O_12_ square plates as well as the assembly of CQDs on Bi_4_Ti_3_O_12_ were carried out via a hydrothermal route. Moreover, the growth process of Bi_4_Ti_3_O_12_ square plates was systematically investigated. The characteristics, photocatalytic performance and photocatalytic mechanism of the as-prepared CQDs/Bi_4_Ti_3_O_12_ composites were systematically investigated and discussed.

## 2. Materials and Methods 

### 2.1. Synthesis of Bi_4_Ti_3_O_12_ Square Plates

A hydrothermal route was used to synthesize Bi_4_Ti_3_O_12_ square plates. In a typical synthesis, 0.004 mol (1.9402 g) of Bi(NO_3_)_3_·5H_2_O was dissolved in 20 mL of 10% v/v dilute nitric acid solution (solution A), 0.003 mol (0.5691 g) of TiCl_4_ was dissolved in 20 mL of deionized water (solution B), and 0.2 mol (8 g) of NaOH was dissolved in 40 mL of deionized water (solution C). The solutions B and C were successively dropped in the solution A slowly with the aid of mild magnetic stirring. The resultant mixture was loaded and sealed into a 100 mL Teflon-lined stainless steel autoclave and subjected to heat treatment at 200 °C. To investigate the growth process of Bi_4_Ti_3_O_12_ square plates, different heat treatment temperatures (3, 6, 12, 24 and 36 h) were carried out. After the autoclave was naturally cooled to room temperature, the produced precipitate was collected by centrifugation, washed several times with deionized water and absolute ethanol, and dried at 60 °C for 12 h.

### 2.2. Assembly of CQDs on the Surface of Bi_4_Ti_3_O_12_ Square Plates

Bi_4_Ti_3_O_12_ square plates synthesized at 200 °C for 24 h via the above hydrothermal route were used to be decorated with CQDs in two steps. The first step was to prepare CQDs suspension using glucose as the carbon source. One gram of glucose was added to 80 mL deionized water, followed by ultrasonic treatment for 30 min and magnetic stirring for another 30 min. The glucose solution was then loaded into a Teflon-lined stainless steel autoclave (100 mL) and subjected to heat treatment at 180 °C for 4 h. After the autoclave was naturally cooled to room temperature, the solution was filtered through a millipore filter paper two times. The obtained reddish-brown solution was CQDs suspension. The second step was to assemble CQDs on the surface of Bi_4_Ti_3_O_12_ square plates. In a typical assembly process, 0.1 g of Bi_4_Ti_3_O_12_ plates was added into 70 mL of deionized water and ultrasonicated for 30 min. Ten milliliters of the CQDs suspension was added drop by drop into the Bi_4_Ti_3_O_12_ suspension and magnetically stirred for 1 h. The resultant mixture was loaded into a 100 mL Teflon-lined stainless steel autoclave and subjected to heat treatment at 130 °C for 4 h. After cooling to room temperature naturally, the precipitate was collected by centrifugation and dried at 60 °C for 12 h. The final product was obtained as the CQDs@Bi_4_Ti_3_O_12_ composite. According to this procedure, several composite samples with different CQDs contents were prepared by adding different volumes of CQDs solution (5, 10, 15, and 20 mL) into the precursor Bi_4_Ti_3_O_12_ suspension, and the corresponding samples were designated as 5CQDs@Bi_4_Ti_3_O_12_, 10CQDs@Bi_4_Ti_3_O_12_, 15CQDs@Bi_4_Ti_3_O_12_, and 20CQDs@Bi_4_Ti_3_O_12_.

### 2.3. Sample Characterization

The crystal structure of the samples was identified by x-ray powder diffraction (XRD) with Cu Kα radiation (λ = 0.15406 nm) on a D8 Advance x-ray diffractometer (Bruker AXS, Karlsruhe, Germany). Scanning electron microscopy (SEM) and transmission electron microscopy (TEM) were used to investigate the morphology and microstructure of the samples. The SEM and TEM investigation was performed on a JSM-6701F scanning electron microscope (JEOL Ltd., Tokyo, Japan) and a JEM-1200EX transmission electron microscope (JEOL Ltd., Tokyo, Japan). Energy-dispersive x-ray spectroscopy (EDS) attached to TEM was used to analyze the chemical composition and elemental distribution of the samples. The optical absorption and bandgap energy of the samples were characterized by UV-vis diffuse reflectance spectroscopy (DRS) on a TU-1901 double beam UV-vis spectrophotometer (Beijing Purkinje General Instrument Co. Ltd., Beijing, China). X-ray photoelectron spectroscopy (XPS) measurement was carried out on a PHI-5702 multi-functional x-ray photoelectron spectrometer (Physical Electronics, hanhassen, MN, USA) to reveal the composition and chemical states of elements in the samples. Fourier transform infrared spectroscopy (FTIR) analysis was performed on a Spectrum Two FTIR spectrophotometer (PerkinElmer, Waltham, MA, USA) in the range of 500–4000 cm^−1^ using KBr powder. A RF-6000 fluorescence spectrophotometer (Shimadzu, Kyoto, Japan) was used to measure the photoluminescence (PL) spectra of the samples with an excitation wavelength of 320 nm.

### 2.4. Photocatalytic Testing

Simulated sunlight emitted by a 200 W xenon lamp (300 < λ < 2500 nm) and visible light generated by a 200 W halogen-tungsten lamp (λ > 400 nm) were separately used as the light source to evaluate the photocatalytic activity of the samples toward the degradation of rhodamine B (RhB) in aqueous solution. Such as in a typical photocatalytic experiment, 0.2 g of the photocatalyst was loaded into 100 mL of 5 mg·L^−1^ RhB aqueous solution. The mixture was put into the photoreactor and magnetically stirred in the dark for 30 min to determine the adsorption of RhB onto the photocatalyst surface. During the photocatalytic process, a circulating water cooling system was used to cool the photoreactor in order to maintain the reaction solution at room temperature (~21 °C). The residual concentration of RhB was monitored every 30 min. To achieve this, 2.5 mL of the reaction solution was sampled from the photoreactor and centrifugated to remove the photocatalyst. The residual concentration of RhB was determined by measuring the absorbance of the reaction solution on a UV–vis spectrophotometer at λ = 554 nm. The degradation percentage of RhB is defined as follows: *D*% = (1 − *C_t_*/*C*_0_) × 100%, where *C*_0_ and *C_t_* represent the initial and residual RhB concentrations after photocatalysis for *t* min.

### 2.5 Electrochemical Measurement

A three-electrode cell configuration was used for the transient photocurrent response and electrochemical impedance spectroscopy (EIS) measurements of the samples on a CST 350 electrochemical workstation (Wuhan Corrtest Instruments Co. Ltd., Wuhan, China) [50,51]. A platinum foil electrode and a standard calomel electrode (SCE) were used as the counter electrode and reference electrode, respectively. The working electrode was prepared following the procedure described in the literature [51]. Stoichiometric amounts of the photocatalyst (15 mg), acetylene black (0.75 mg) and polyvinylidene fluoride (PVDF, 0.75 mg) were uniformly mixed into 1 mL 1-methyl-2-pyrrolidione (NMP) that acted as the dispersant. The paste mixture was homogeneously coated onto fluorine-doped tin oxide (FTO) glass substrate (effective area: 1 × 1 cm^2^). After drying at 60 °C for 5 h, the final working electrode was obtained. The electrolyte used in this study is a Na_2_SO_4_ aqueous solution with a concentration of 0.1 mol·L^−1^. Simulated sunlight emitted from a 200 W xenon lamp was used as the light source. The transient photocurrent response measurement was performed at a bias potential of 0.2 V. The EIS measurement was performed by imposing a small sinusoidal voltage of 5 mV amplitude over the frequency range from 100 kHz to 0.01 Hz. To investigate the flat band potential of Bi_4_Ti_3_O_12_, the EIS was measured at different applied potentials in the dark.

## 3. Results and Discussion

### 3.1. Growth Process of Bi_4_Ti_3_O_12_ Square Plates

**Figure 1:**
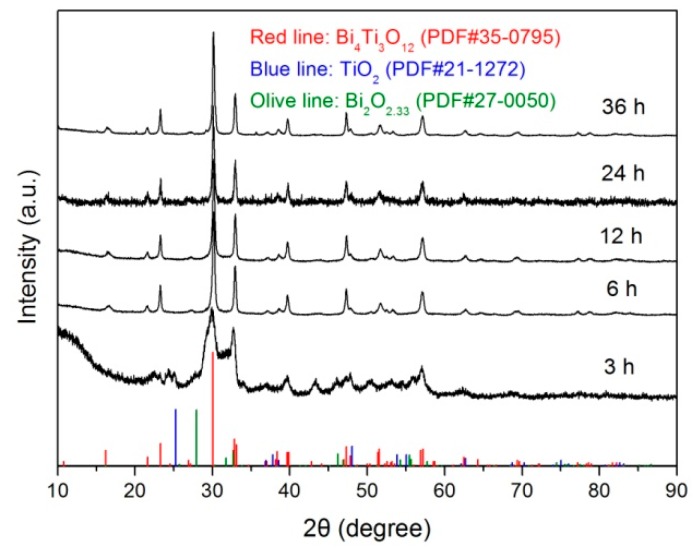
XRD patterns of Bi_4_Ti_3_O_12_ samples prepared at 200 °C with different reaction times (*t* = 3, 6, 12, 24, and 36 h).

Figure 1 shows the XRD patterns of Bi_4_Ti_3_O_12_ samples prepared at 200 °C with different reaction times (*t* = 3, 6, 12, 24, and 36 h). It is seen that the sample obtained at *t* = 3 h consists of a mixture of phases including Bi_4_Ti_3_O_12_ (PDF#35-0795), Bi_2_O_3_ (PDF#27-0050) and anatase TiO_2_ (PDF#21-1272). Moreover, a broad peak at around 2θ = 10° is observed on the XRD pattern, which arises from the amorphous Bi(OH)_3_ and Ti(OH)_4_ precipitates hydrolyzed from Bi(NO_3_)_3_ and TiCl_4_. When the reaction time is increased up to 6 h, the diffraction peaks related to Bi_2_O_3_, TiO_2_ and amorphous substances disappear. All the diffraction peaks can be indexed according to the standard diffraction pattern of PDF#35-0795, implying the formation of a single Bi_4_Ti_3_O_12_ orthorhombic phase. With prolonging the reaction time, the prepared samples maintain a pure Bi_4_Ti_3_O_12_ orthorhombic phase without the appearance of other impurities.

**Figure 2:**
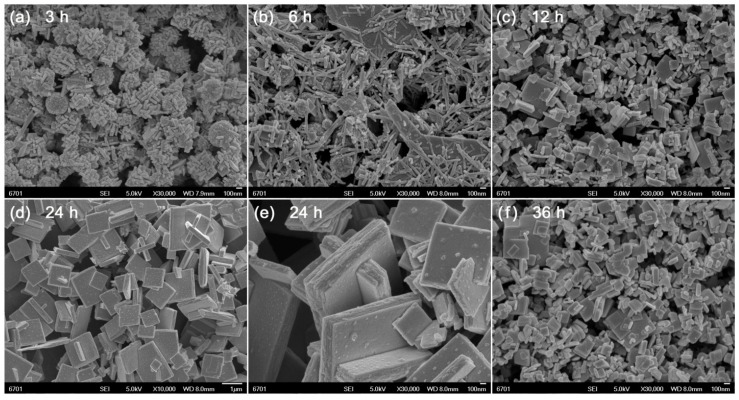
SEM images of Bi_4_Ti_3_O_12_ samples prepared at different reaction times (*t* = 3, 6, 12, 24, and 36 h).

Figure 2 shows the SEM images of Bi_4_Ti_3_O_12_ samples prepared at different reaction times (*t* = 3, 6, 12, 24, and 36 h). As shown in Figure 2a, the product obtained at 3 h reaction time is composed of nanorods, spherical nanoparticles and amorphous aggregates, which are characterized as Bi_4_Ti_3_O_12_, Bi_2_O_3_, TiO_2_, and Bi(OH)_3_/Ti(OH)_4_ precipitates. At 6 h reaction time (Figure 2b), the Bi_2_O_3_/TiO_2_ spherical nanoparticles and Bi(OH)_3_/Ti(OH)_4_ precipitates are crystallized into Bi_4_Ti_3_O_12_ nanorods. With further increasing the reaction time up to 12 h (Figure 2c), the formed Bi_4_Ti_3_O_12_ nanorods gradually grow into brick-like nanoparticles. When the reaction time is increased up to 24 h (Figure 2d,e), it brings about the synthesis of Bi_4_Ti_3_O_12_ square plates with an average lateral size of ~1 μm and thickness of ~200 nm. However, too long a reaction time leads to partial dissolution of the square plates into small-sized brick-like nanoparticles, as shown in Figure 2f.

**Figure 3:**
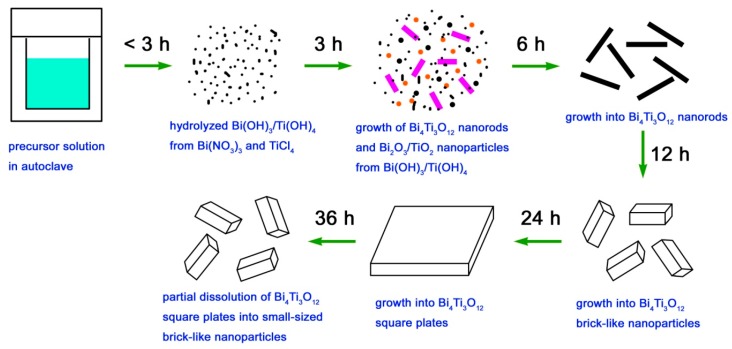
Schematic illustration of the formation process of Bi_4_Ti_3_O_12_ square plates.

Based on the XRD and SEM results, the formation process of Bi_4_Ti_3_O_12_ square plates is schematically illustrated in Figure 3. The ‘‘dissolution–crystallization’’ mechanism can be used to describe the hydrothermal formation of Bi_4_Ti_3_O_12_ square plates [52]. In the precursor solution, Bi(NO_3_)_3_·and TiCl_4_ were hydrolyzed into Bi(OH)_3_ and Ti(OH)_4_ hydroxides with the aid of NaOH. At high temperature and pressure environments, the Bi(OH)_3_ and Ti(OH)_4_ precipitates are to dissolve and form ion groups under the action of NaOH that serves as the mineralizer. In the locally formed supersaturated fluid region, Bi_4_Ti_3_O_12_ nanorods are crystallized by nucleation, precipitation, dehydration, and growth. Simultaneously, Bi_2_O_3_ and TiO_2_ could also be formed in this process. With the hydrothermal reaction going on, Bi_2_O_3_ and TiO_2_ will dissolve again to form ion groups under the attack of NaOH, which will then subsequently crystallize into Bi_4_Ti_3_O_12_ nanorods. The formation of Bi_4_Ti_3_O_12_ crystals can be simply described by Equations (1) and (2). With increasing the reaction time, Bi_4_Ti_3_O_12_ nanorods gradually grow into brick-like nanoparticles and then into square plates. The formation of Bi_4_Ti_3_O_12_ square plates can be explained as a result of the reduction of the overall surface energy. It is noted that the growth process of Bi_4_Ti_3_O_12_ square plates is simultaneously accompanied by their dissolution process. After a relatively long reaction time, the dissolution process could predominate over the growth process, and as a result, Bi_4_Ti_3_O_12_ square plates are etched into small-sized brick-like nanoparticles.

4Bi(OH)_3_ + 3Ti(OH)_4_ → Bi_4_Ti_3_O_12_ + 12H_2_O (1)

2Bi_2_O_3_ + 3TiO_2_ → Bi_4_Ti_3_O_12_ (2)

### 3.2. CQDs-modified Bi_4_Ti_3_O_12_ Square Plates

**Figure 4:**
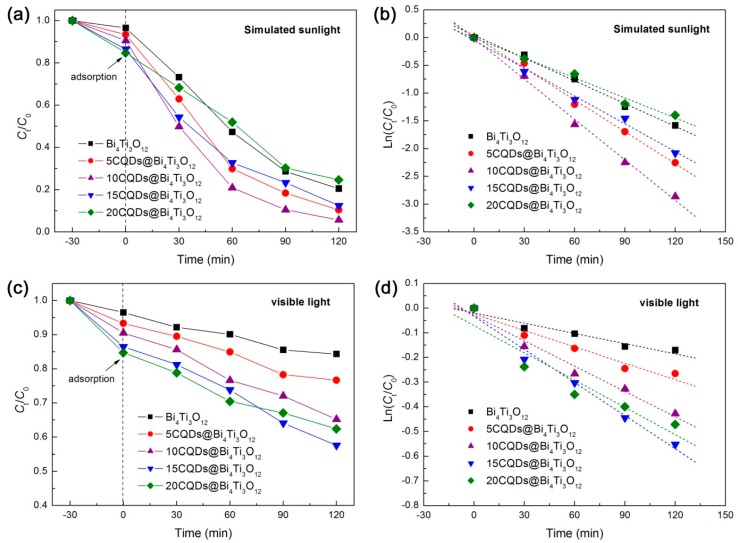
(**a**) Time-dependent photocatalytic degradation of RhB over Bi_4_Ti_3_O_12_ and CQDs@Bi_4_Ti_3_O_12_ composites with different CQDs contents under simulated sunlight irradiation. (**b**) Degradation kinetic plots of RhB over the samples under simulated sunlight irradiation. (**c**) Time-dependent photocatalytic degradation of RhB over the samples under visible light irradiation. (**d**) Degradation kinetic plots of RhB over the samples under visible light irradiation.

The photocatalytic activity of the as-prepared CQDs@Bi_4_Ti_3_O_12_ composites was evaluated by the degradation of RhB in aqueous solution separately under the irradiation of simulated sunlight (300 < λ < 2500 nm) and visible light (λ > 400 nm). Before the photocatalytic degradation experiment, the adsorption of RhB onto bare Bi_4_Ti_3_O_12_ and CQDs@Bi_4_Ti_3_O_12_ composites was measured in the dark at 30 min of contact time and is obtained as 3.5‒15.3%. It is observed that the RhB adsorption gradually increases with increasing the CQDs content, which is ascribed to the enhanced dye adsorption of CQDs. Generally, an appropriate increase in the dye adsorption is conducive to photocatalysis. The time-dependent photocatalytic degradation of RhB under simulated sunlight irradiation is shown in Figure 4a. The degradation percentage of RhB after 120 min of photocatalysis is given in Table 1. It is seen that the decoration of an appropriate amount of CQDs on Bi_4_Ti_3_O_12_ leads to a significantly enhanced photocatalytic activity. The highest photocatalytic performance is observed for the 10CQDs@Bi_4_Ti_3_O_12_ composite prepared by adding 10 mL CQDs solution, and the degradation percentage of RhB reaches 94.3%. However, decoration of excessive CQDs on Bi_4_Ti_3_O_12_ could reduce the light absorption of Bi_4_Ti_3_O_12_, and thus, give rise to a decrease in the photocatalytic activity. To further compare the photocatalytic activity between the samples, the degradation kinetics of RhB were investigated. As shown in Figure 4b, the plots of Ln(*C_t_*/*C*_0_) *vs* irradiation time *t* can be well modeled using the pseudo-first-order kinetic equation: Ln(*C_t_*/*C*_0_) = −*k*_app_*t*, where *k*_app_ is the apparent first-order reaction rate constant [53]. The obtained value of *k*_app_ from the slope of the regression lines is given in Table 1. It is seen that the optimal composite 10CQDs@Bi_4_Ti_3_O_12_ manifests a photocatalytic activity ca. 1.8 times higher than that of bare Bi_4_Ti_3_O_12_. Figure 4c shows the visible-light photocatalytic degradation of RhB over bare Bi_4_Ti_3_O_12_ and CQDs@Bi_4_Ti_3_O_12_ composites, and Figure 4d presents the plots of Ln(*C_t_*/*C*_0_) *vs* irradiation time *t*. The obtained degradation percentage after photocatalytic reaction for 120 min and reaction rate constant *k*_app_ are summarized in Table 1. Photocatalyzed by bare Bi_4_Ti_3_O_12_, only 15.7% of the dye is observed to be degraded, which is mainly ascribed to the dye adsorption and dye-photosensitized degradation. This indicates that bare Bi_4_Ti_3_O_12_ exhibits poor photocatalytic activity under visible light irradiation. When compared with bare Bi_4_Ti_3_O_12_, the CQDs@Bi_4_Ti_3_O_12_ composites manifest a greatly enhanced visible-light photocatalytic activity. In particular, the 15CQDs@Bi_4_Ti_3_O_12_ composite photocatalyzes 42.4% removal of the dye and displays a photocatalytic activity ca. 3.2 times as large as that of bare Bi_4_Ti_3_O_12_ according to the reaction rate constants.

**Figure 5:**
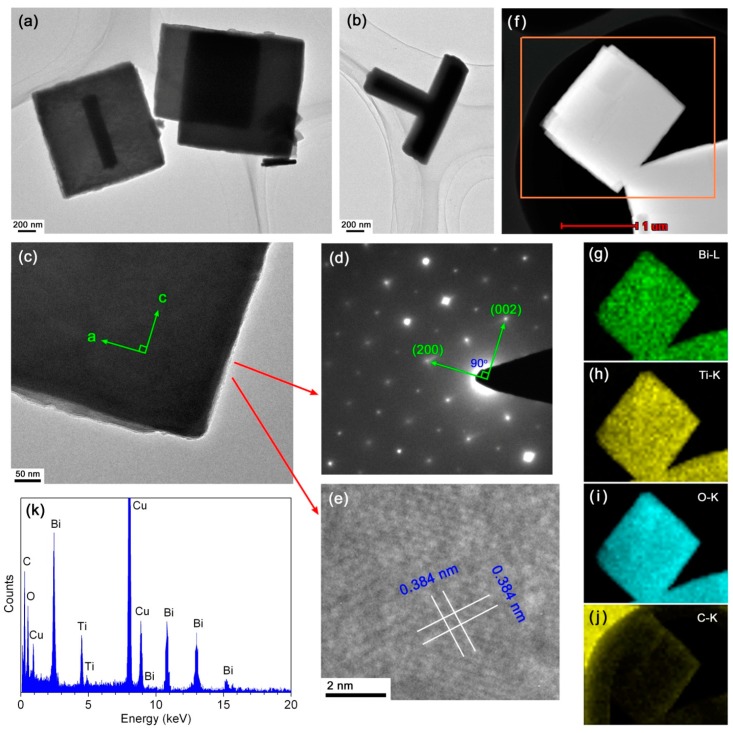
(**a**‒**c**) TEM images of 10CQDs@Bi_4_Ti_3_O_12_. (**d**) and (**e**) SAED pattern and HRTEM image obtained from the Bi_4_Ti_3_O_12_ square plate of (c), respectively. (**f**) DF-STEM image of 10CQDs@Bi_4_Ti_3_O_12_. (**g**–**j**) The corresponding energy-dispersive x-ray elemental mapping images of the region marked by orange rectangle in (f). (**k**) EDS spectrum of 10CQDs@Bi_4_Ti_3_O_12_.

Further investigation was specially carried out on the 10CQDs@Bi_4_Ti_3_O_12_ composite prepared with 10 mL CQDs solution with the aim of revealing its microstructure and enhanced photocatalytic mechanism. Figure 5a–c shows the TEM images of 10CQDs@Bi_4_Ti_3_O_12_. It is seen that Bi_4_Ti_3_O_12_ has a geometrical morphology of square plates. Occasionally, the square plates are assembled into a T-shape structure, as shown in Figure 5b. Figure 5d shows the selected area electron diffraction (SAED) pattern obtained from the Bi_4_Ti_3_O_12_ square plate of Figure 5c. The diffraction spots are regularly and periodically arranged, which can be indexed to the [010] zone axis of Bi_4_Ti_3_O_12_ orthorhombic structure. The a-axis and b-axis are parallel to the lateral side of the square plate. The highly exposed plane of the square plate is identified to be the (010) facet. This crystallographic orientation of the Bi_4_Ti_3_O_12_ square plate is further confirmed by the high resolution TEM (HRTEM) image, as shown in Figure 5e. The lattice fringes with d-spacing of 0.384 nm are observed to be parallel or perpendicular to the diagonal direction of the square plate and match well with the (202) crystal plane of Bi_4_Ti_3_O_12_ orthorhombic phase. Although the TEM/HRTEM images and SAED pattern cannot show the presence of CQDs on Bi_4_Ti_3_O_12_ plates, other methods can be used to determine the formation of CQDs@Bi_4_Ti_3_O_12_ composites. Figure 5f shows the dark-field scanning TEM (DF-STEM) image of the 10CQDs@Bi_4_Ti_3_O_12_ composite, and Figure 5g–j presents the corresponding energy-dispersive x-ray elemental mapping images of the region marked by the orange rectangle in Figure 5f. Besides the elements of Bi, Ti and O which constitute Bi_4_Ti_3_O_12_ square plates, C element is also observed to be uniformly distributed throughout the plates. The elemental mapping images confirm the uniform assembly of CQDs on the surface of Bi_4_Ti_3_O_12_ plates. The chemical composition of 10CQDs@Bi_4_Ti_3_O_12_ was further investigated by EDS spectrum, as shown in Figure 5k. It is obvious that the 10CQDs@Bi_4_Ti_3_O_12_ sample is composed of Bi, Ti, O, and C elements. The observed Cu signal on the EDS spectrum could arise from the microgrid used for supporting the sample in the TEM experiment [54]. The Bi/Ti atomic ratio obtained from the EDS spectrum is very close to that in the Bi_4_Ti_3_O_12_ phase. However, the EDS spectrum shows a lower O content with respect to oxygen in Bi_4_Ti_3_O_12_, which could be ascribed to non-sensitivity of EDS to light elements like O [54].

**Figure 6:**
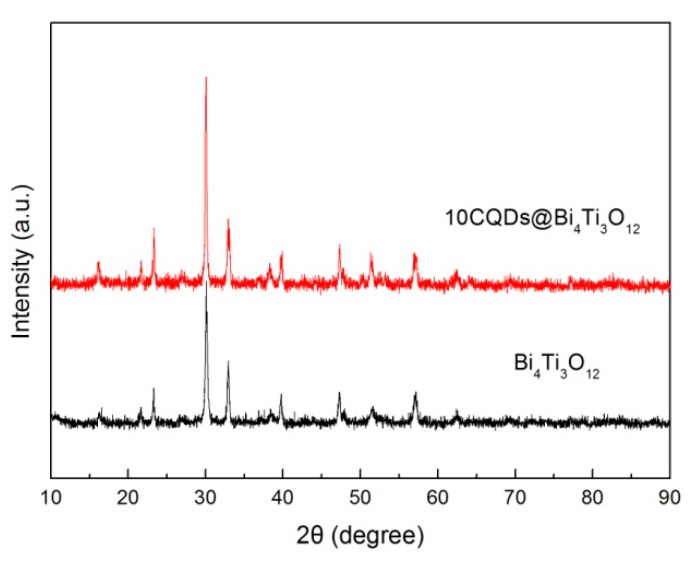
XRD patterns of Bi_4_Ti_3_O_12_ and 10CQDs@Bi_4_Ti_3_O_12_.

Figure 6 shows the XRD patterns of Bi_4_Ti_3_O_12_ and 10CQDs@Bi_4_Ti_3_O_12_. It is seen that the diffraction peaks of 10CQDs@Bi_4_Ti_3_O_12_ are very similar to those of bare Bi_4_Ti_3_O_12_, implying that Bi_4_Ti_3_O_12_ undergoes no structural change when assembled with CQDs. No obvious diffraction signals assignable to CQDs are detected in the XRD pattern. The possible reason may be that CQDs have extremely weak x-ray diffraction due to the lack of long-range-ordered structure, and moreover, CQDs account for only a small fraction of the total mass of the 10CQDs@Bi_4_Ti_3_O_12_ composite.

**Figure 7:**
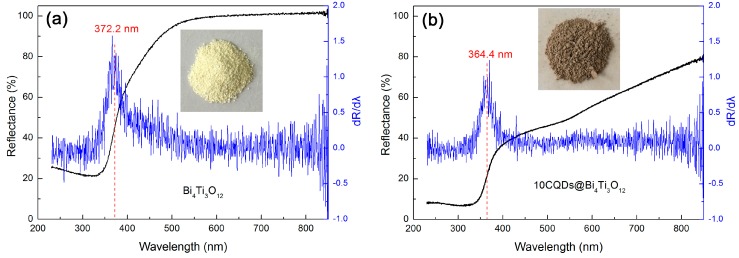
UV-vis DRS spectra, first derivative curves of the UV-vis DRS spectra and digital images of (**a**) Bi_4_Ti_3_O_12_ and (**b**) 10CQDs@Bi_4_Ti_3_O_12_.

Figure 7a,b shows the UV-vis DRS spectra of Bi_4_Ti_3_O_12_ and 10CQDs@Bi_4_Ti_3_O_12_. A significantly enhanced light absorption in the wavelength range of 400‒850 nm is observed for the 10CQDs@Bi_4_Ti_3_O_12_ composite when compared with bare Bi_4_Ti_3_O_12_. The digital images of the samples separately inserted in Figure 7a,b demonstrate that bare Bi_4_Ti_3_O_12_ is cream white, whereas 10CQDs@Bi_4_Ti_3_O_12_ manifests a gray color. The deepening of the apparent color for 10CQDs@Bi_4_Ti_3_O_12_ further confirms its enhanced visible-light absorption. This implies that the composite can make more efficient utilization of sunlight during photocatalysis. The first derivative curves of the UV-vis DRS spectra are used to determine the absorption edge of the samples [55]. As seen from Figure 7a,b, Bi_4_Ti_3_O_12_ and 10CQDs@Bi_4_Ti_3_O_12_ exhibit an absorption edge of 372.2 and 364.4 nm, respectively. Based on the absorption edge, the bandgap energy (*E*_g_) can be derived from the relationship *E*_g_ = 1240/λ_abs_ (λ_abs_ represents the absorption edge wavelength), and is obtained as 3.33 eV for bare Bi_4_Ti_3_O_12_ and 3.40 eV for 10CQDs@Bi_4_Ti_3_O_12_. The slight increase in the *E*_g_ of the composite could be ascribed to the interaction between Bi_4_Ti_3_O_12_ plates and CQDs.

**Figure 8:**
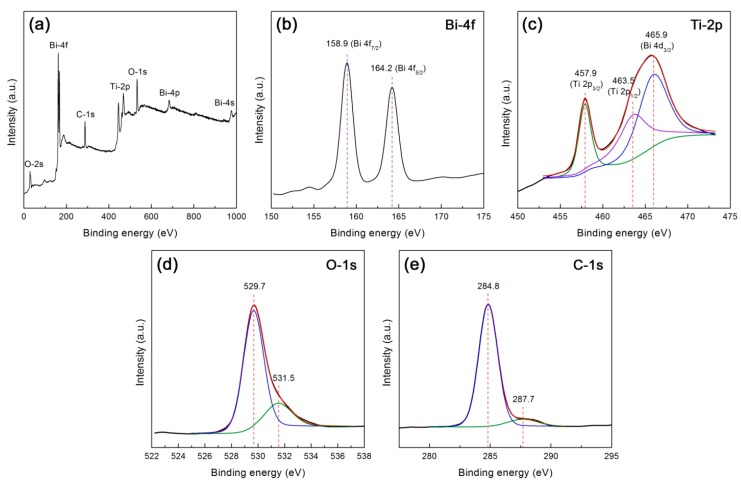
XPS spectra of 10CQDs@Bi_4_Ti_3_O_12_. (**a**) Survey scan spectrum; (**b**)–(**e**) High resolution XPS spectra of Bi 4f, Ti 2p, O 1s, and C 1s, respectively.

XPS analysis was carried out to reveal the composition and element chemical states of the 10CQDs@Bi_4_Ti_3_O_12_ composite. Figure 8a shows the XPS survey scan spectrum, revealing the existence of Bi, Ti, O, and C elements in the composite. Figure 8b–e illustrates the high resolution XPS spectra of Bi 4f, Ti 2p, O 1s, and C 1s, respectively. As seen from Figure 8b, the Bi 4f XPS spectrum shows two sharp peaks at 158.9 and 164.2 eV, which are attributed to the Bi 4f_7/2_ and Bi 4f_5/2_ binding energies, respectively [33,56,57]. The Ti 2p XPS spectrum shown in Figure 8c can be deconvoluted into three peaks at 457.9, 463.5 and 465.9 eV, which correspond to the binding energies of Ti 2p_3/__2,_ Ti 2p_1/2_ and Bi 4d_3/2_ respectively [56,57]. The Bi 4f and Ti 2p binding energy peaks suggest that bismuth species exists in +3 oxidation state and titanium species exhibits +4 oxidation state. The existence of other oxidation states can be excluded since no additional binding energy peaks are detected on the Bi 4f and Ti 2p XPS spectra. On the O 1s XPS spectrum (Figure 8d), the binding energy at 529.7 eV is attributed to the crystal lattice oxygen of Bi_4_Ti_3_O_12_, whereas the binding energy at 531.5 eV could arise from chemisorbed oxygen species [58,59]. The C 1s XPS spectrum (Figure 8e) presents two peaks at 284.8 and 287.7 eV. The peak at 284.8 eV arises from the C–C sp^2^-hybridized carbon of CQDs and adventitious carbon that is used to calibrate the binding energy scale [59,60]. The peak at 287.7 eV is attributed to the presence of C–O–C or C=O [60]. 

**Figure 9:**
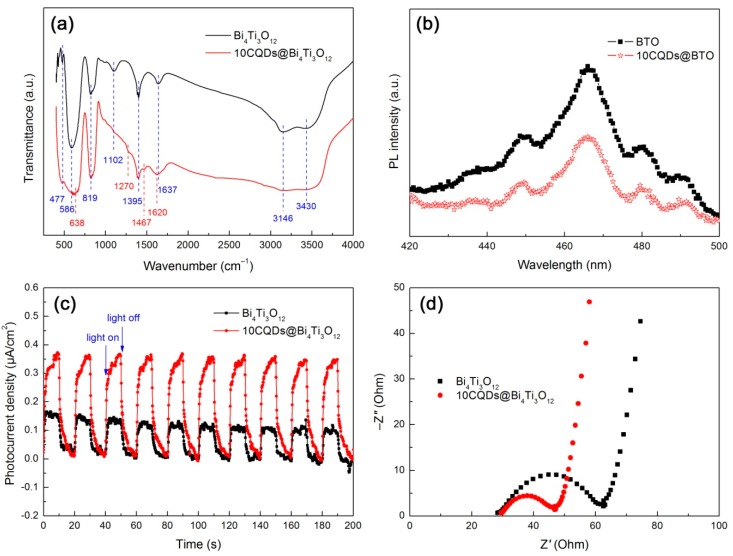
FTIR spectra (**a**), PL spectra (**b**), transient photocurrent response curves under intermittent irradiation of simulated sunlight, (**c**) and Nyquist plots of the EIS spectra under simulated sunlight irradiation (**d**) of Bi_4_Ti_3_O_12_ and 10CQDs@Bi_4_Ti_3_O_12_.

The functional groups of Bi_4_Ti_3_O_12_ and 10CQDs@Bi_4_Ti_3_O_12_ were analyzed by FTIR spectra, as shown in Figure 9a. The broad bands detected at 3430 and 1637 cm^−1^ correspond to the stretching and bending vibrations of water molecules absorbed on the surface of the samples, respectively [61]. The peaks observed at 1102 and 1395 cm^−1^ account for the C–OH stretching and O–H in-plane deformation vibrations of alcohols left on the samples during their washing process. The broad absorption band at around 3146 cm^−1^ is assigned to the N–H stretching vibration of the NH^3+^ group [62]. The strong peak at 477 cm^−1^ and weak peak at 586 cm^−1^ are characterized as the Ti–O stretching vibration. The characteristic stretching vibration of Bi–O is observed at 819 cm^−1^ [63,64]. This implies the formation of the Bi_4_Ti_3_O_12_ orthorhombic phase and its crystal structure undergoes no destruction when decorated with CQDs. Additional peaks at 638, 1270, 1467, and 1620 cm^−1^ are detected for the 10CQDs@Bi_4_Ti_3_O_12_ composite. The former two peaks (638 and 1270 cm^−1^) are ascribed to the C–H deformation vibration and the latter two peaks (1467 and 1620 cm^−1^) can be attributed to the C–C stretching vibration [65]. These absorption peaks suggest that CQDs are assembled on the surface of Bi_4_Ti_3_O_12_ plates.

To reveal the separation and transfer behavior of photogenerated electrons and holes, PL, photocurrent response and EIS analyses were carried out. Figure 9b shows the PL spectra of Bi_4_Ti_3_O_12_ and 10CQDs@Bi_4_Ti_3_O_12_ measured at an excitation wavelength of 320 nm. Both samples clearly display the PL emission peaks at 448, 466, 480, and 491 nm, which arise due to the recombination of photogenerated electron/hole pairs. When compared with bare Bi_4_Ti_3_O_12_, the 10CQDs@Bi_4_Ti_3_O_12_ composite exhibits the PL emission peaks with relatively lower intensity, implying a decreased electron/hole recombination. The efficient separation of electron/hole pairs occurring in the composite can be explained as a result of the electron transfer from the conduction band (CB) of Bi_4_Ti_3_O_12_ to CQDs.

Figure 9c shows the transient photocurrent response curves of Bi_4_Ti_3_O_12_ and 10CQDs@Bi_4_Ti_3_O_12_ measured under intermittent irradiation of simulated sunlight with 10 min irradiation followed by 10 min intervals. When the simulated sunlight is turned off, a photocurrent density of ca. 0.10–0.16 and 0.34–0.37 μA·cm^−2^ is observed for bare Bi_4_Ti_3_O_12_ and 10CQDs@Bi_4_Ti_3_O_12_, respectively. The photocurrent density for both samples immediately drops almost to zero when the light is turned off. This effect appears to be very reproducible upon alternately turning on and off the light. The transient photocurrent measurement clearly demonstrates that the 10CQDs@Bi_4_Ti_3_O_12_ composite exhibits a more efficient separation of photogenerated electron/hole pairs than bare Bi_4_Ti_3_O_12_. This result is further confirmed by the Nyquist plots of the EIS spectra, as shown in Figure 9d. It is clearly seen that the Nyquist plots of both samples display a semicircle (high-frequency region) followed by a straight line (low-frequency region). However, a smaller semicircle diameter is observed for the composite. It is well established that the semicircle diameter of the Nyquist plots is correlated with the charge-transfer resistance at the electrode/electrolyte interface, and a smaller diameter suggests a smaller charge-transfer resistance [66,67]. The EIS spectrum analysis elucidates an enhanced electron/hole pair separation and interface charge transfer under irradiation of simulated sunlight for the 10CQDs@Bi_4_Ti_3_O_12_ composite.

**Figure 10:**
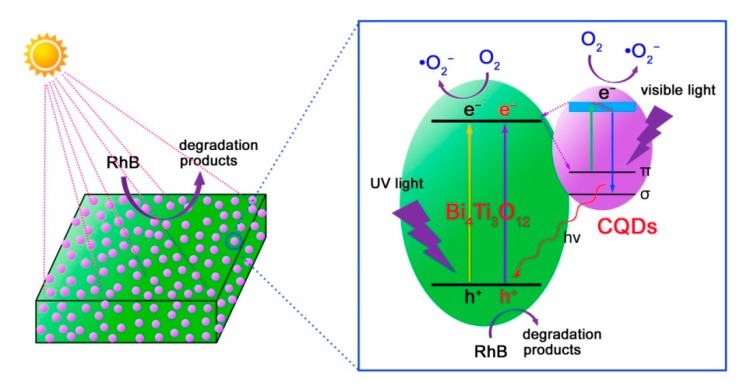
Schematic illustration of the photocatalytic mechanism of CQDs@Bi_4_Ti_3_O_12_ for the dye degradation under simulated sunlight irradiation.

Based on the aforementioned experimental results and analyses, a possible photocatalytic mechanism of CQDs@Bi_4_Ti_3_O_12_ toward the dye degradation is proposed, as schematically illustrated in Figure 10. Under simulated sunlight irradiation, electrons are excited from the valence band (VB) of Bi_4_Ti_3_O_12_ to its CB, thus creating electron/hole pairs. Simultaneously, the electronic excitation process also occurs in CQDs, i.e., electrons in the π orbital or σ orbital of CQDs are excited to a high-energy state such as the lowest unoccupied molecular orbital (LUMO) [19]. The photoexcited CQDs are excellent electron donors and electron acceptors. As a result, the CB electrons in Bi_4_Ti_3_O_12_ will transfer to the π orbital or σ orbital of CQDs. Conversely, the photoexcited electrons in CQDs can be also transferred to the CB of Bi_4_Ti_3_O_12_. The interesting electron transfer process can efficiently suppress the recombination of photogenerated electron/hole pairs in Bi_4_Ti_3_O_12_. Consequently, more photogenerated carriers are able to participate in the photocatalytic reactions, thus leading to an increased photocatalytic performance of the CQDs@Bi_4_Ti_3_O_12_ composites under simulated sunlight irradiation. It is noted that the Bi_4_Ti_3_O_12_ square plates have a bandgap of 3.33 eV, implying that bare Bi_4_Ti_3_O_12_ plates exhibit a poor photocatalytic activity under visible light irradiation. However, when CQDs are decorated on Bi_4_Ti_3_O_12_, the obtained CQDs@Bi_4_Ti_3_O_12_ composites manifest an obviously enhanced visible-light photocatalytic activity toward the dye degradation, as shown in Figure 4c,d. This phenomenon can be attributed to the following reasons. Under visible light irradiation, the π electrons in CQDs could be excited to the LUMO. When the electrons fall back to the σ orbital, an up-conversion photoluminescence is emitted [19]. The up-conversion photoluminescence could have photon energy larger than the bandgap energy of Bi_4_Ti_3_O_12_ and would induce the electron excitation from the VB to the CB of Bi_4_Ti_3_O_12_. The PL-induced electrons and holes are able to participate in the photocatalytic reactions. On the other hand, the photoexcited electrons in CQDs could also participate in the photocatalytic reactions. The two factors collectively result in the enhanced visible-light photocatalytic activity of the CQDs@Bi_4_Ti_3_O_12_ composites.

**Figure 11:**
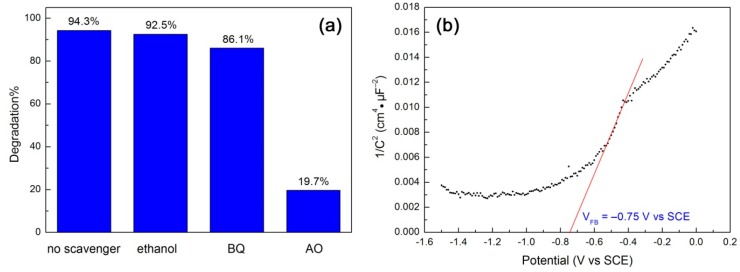
(**a**) Effect of ethanol, BQ and AO on the degradation percentage of RhB at 120 min of photocatalysis. (**b**) Mott–Schottky plot of Bi_4_Ti_3_O_12_ square plates obtained at 5000 Hz.

Reactive species trapping experiments were carried out to reveal their role in the degradation of RhB over 10CQDs@Bi_4_Ti_3_O_12_. In this study, ethanol, benzoquinone (BQ) and ammonium oxalate (AO) were used as the scavengers of hydroxyl (•OH), superoxide (•O_2_^−^) and photogenerated h^+^, respectively [68]. Figure 11a shows the effect of ethanol (5 mL), BQ (0.1 mmol) and AO (0.1 mmol) on the degradation percentage of RhB at 120 min of photocatalysis. It is seen that the addition of ethanol to the reaction solution has almost no effect on the dye degradation, implying a minor or negligible role of •OH in the dye degradation. The addition of BQ leads to a slight suppression of the dye degradation. This suggests that the role of •O_2_^−^ cannot be neglected, but it plays only a slight role in the photocatalytic reaction. With the addition of AO, however, the dye degradation is significantly suppressed and only 19.7% of RhB is observed to be degraded. This indicates that the photogenerated h^+^ is the dominant active species responsible for the dye degradation. To reveal the CB and VB potentials of Bi_4_Ti_3_O_12_ square plates, we carried out the Mott–Schottky measurement according to the method described in the literature [69,70,71], as shown in Figure 11b. By extrapolating the linear portion of the Mott–Schottky plot to the V axis, the flat band potential (V_FB_) is obtained as –0.75 V vs SCE. The SCE potential can be converted to the potential against a normal hydrogen electrode (NHE) using V(NHE) = V(SCE) + 0.059pH + 0.242 (here pH = 7) [71]. The positive slope of the Mott–Schottky plot suggests that Bi_4_Ti_3_O_12_ exhibits an n-type semiconductivity. Assuming that the gap between the flat band potential and the CB edge is negligible for an n-type semiconductor, the CB potential of Bi_4_Ti_3_O_12_ square plates is therefore estimated as –0.10 V vs NHE. Their VB potential is obtained as +3.23 V vs NHE using V_VB_ = V_CB_ − *E_g_* (*E_g_* = 3.33 V). It is noted that the VB potential of Bi_4_Ti_3_O_12_ is more positive than the redox potentials of H_2_O/•OH (+2.38 V vs NHE) and OH^−^/•OH (+1.99 V vs NHE) [72]. However, the reactive species trapping experiments demonstrate that •OH plays a negligible role in the dye degradation. To further confirm this, we also measured •OH radicals by PL spectroscopy using terephthalic acid as a probe of •OH, as described in the literature [72]. Nevertheless, no •OH radicals are detected in the 10CQDs@Bi_4_Ti_3_O_12_ photocatalytic system. The generation of minor •O_2_^−^ radicals could be derived from the reaction of adsorbed O_2_ molecules with the photoexcited electrons in CQDs or photogenerated electrons at higher excited states of Bi_4_Ti_3_O_12_.

## 4. Conclusions

A hydrothermal route was used to synthesize Bi_4_Ti_3_O_12_ square plates with an average lateral size of ~1 μm and average thickness of ~200 nm. The growth process of the square plates was systematically investigated. CQDs derived from glucose were assembled on the surface of Bi_4_Ti_3_O_12_ square plates via a hydrothermal route. Compared to bare Bi_4_Ti_3_O_12_, the as-prepared CQDs@Bi_4_Ti_3_O_12_ composites with an appropriate amount of CQDs manifest an increased photocatalytic performance for the RhB degradation under irradiation of simulated sunlight and visible light. The optimal CQDs@Bi_4_Ti_3_O_12_ composite photocatalyzes 94.3% removal of the dye under simulated sunlight irradiation, demonstrating a photocatalytic activity ca. 1.8 times higher than that of bare Bi_4_Ti_3_O_12_. The enhanced photocatalytic performance of CQDs-modified Bi_4_Ti_3_O_12_ is mainly attributed to the efficient separation of photogenerated electron/hole pairs and increased visible light absorption. The efficient utilization of visible light during the photocatalysis is manifested in 2 aspects: (1) The visible-light photoexcited electrons in CQDs could participate in the photocatalytic reactions, and (2) additional electron/hole pairs in Bi_4_Ti_3_O_12_ could be generated by the up-conversion photoluminescence emitted from CQDs.

## Figures and Tables

**Table 1:** Degradation percentage of RhB (reaction for 120 min) and apparent first-order reaction rate constant *k*_app_ for Bi_4_Ti_3_O_12_ and CQDs@Bi_4_Ti_3_O_12_ composites under irradiation of simulated sunlight and visible light.

Samples	Simulated Sunlight		Visible Light	
	Degradation (%)	*k*_app_ (min^−1^)	Degradation (%)	*k*_app_ (min^−1^)
Bi_4_Ti_3_O_12_	79.5	0.01368	15.7	0.00139
5CQDs@Bi_4_Ti_3_O_12_	89.5	0.01912	23.3	0.00222
10CQDs@Bi_4_Ti_3_O_12_	94.3	0.02426	34.8	0.00342
15CQDs@Bi_4_Ti_3_O_12_	87.5	0.01667	42.4	0.00447
20CQDs@Bi_4_Ti_3_O_12_	75.3	0.01203	37.6	0.00368

## References

[1] Moroz P., Boddy A., Zamkov M. (2018). Challenges and prospects of photocatalytic applications utilizing semiconductor nanocrystals. Front. Chem..

[2] Di L.J., Yang H., Xian T., Chen X.J. (2017). Enhanced photocatalytic activity of NaBH_4_ reduced BiFeO_3_ nanoparticles for rhodamine B decolorization. Materials.

[3] Xia Y.M., He Z.M., Yang W., Tang B., Lu Y.L., Hu K.J., Su J.B., Li X.P. (2018). Effective charge separation in BiOI/Cu_2_O composites with enhanced photocatalytic activity. Mater. Res. Express.

[4] Wang S.F., Gao H.J., Wei Y., Li Y.W., Yang X.H., Fang L.M., Lei L. (2019). Insight into the optical, color, photoluminescence properties, and photocatalytic activity of the N-O and C-O functional groups decorating spinel type magnesium aluminate. CrystEngComm.

[5] Zangeneh H., Zinatizadeh A.A.L., Habibi M., Akia M., Isa M.H. (2015). Photocatalytic oxidation of organic dyes and pollutants in wastewater using different modified titanium dioxides: A comparative review. J. Ind. Eng. Chem..

[6] Zhao W.H., Wei Z.Q., Zhang L., Wu X.J., Wang X. (2018). Cr doped SnS_2_ nanoflowers: Preparation, characterization and photocatalytic decolorization. Mater. Sci. Semicond. Process..

[7] Ariyanti D., Mills L., Dong J., Yao Y., Gao W. (2017). NaBH_4_ modified TiO_2_: Defect site enhancement related to its photocatalytic activity. Mater. Chem. Phys..

[8] Yan Y.X., Yang H., Zhao X.X., Li R.S., Wang X.X. (2018). Enhanced photocatalytic activity of surface disorder-engineered CaTiO_3_. Mater. Res. Bull..

[9] Tayyebi A., Soltani T., Hong H., Lee B.K. (2018). Improved photocatalytic and photoelectrochemical performance of monoclinic bismuth vanadate by surface defect states (Bi_1−x_VO_4_). J. Colloid Interface Sci..

[10] Dutta D.P., Tyagi A.K. (2016). Facile sonochemical synthesis of Ag modified Bi_4_Ti_3_O_12_ nanoparticles with enhanced photocatalytic activity under visible light. Mater. Res. Bull..

[11] Wang F., Yang H., Zhang Y.C. (2018). Enhanced photocatalytic performance of CuBi_2_O_4_ particles decorated with Ag nanowires. Mater. Sci. Semicond. Proc..

[12] Wang H.L., Zhang L.S., Chen Z.G., Hu J.Q., Li S.J., Wang Z.H., Liu J.S., Wang X.C. (2014). Semiconductor heterojunction photocatalysts: design, construction, and photocatalytic performances. Chem. Soc. Rev..

[13] Guerrero-Araque D., Ramirez-Ortega D., Acevedo-Pena P., Tzompantzi F., Calderon H.A., Gomez R. (2017). Interfacial charge-transfer process across ZrO_2_-TiO_2_ heterojunction and its impact on photocatalytic activity. J. Photochem. Photobiol. A-Chem..

[14] Zeng Y., Chen X.F., Yi Z., Yi Y.G., Xu X.B. (2018). Fabrication of p-n heterostructure ZnO/Si moth-eye structures: Antireflection, enhanced charge separation and photocatalytic properties. Appl. Surf. Sci..

[15] Xia Y., He Z., Su J., Liu Y., Tang B. (2018). Fabrication and photocatalytic property of novel SrTiO_3_/Bi_5_O_7_I nanocomposites. Nanoscale Res. Lett..

[16] Di L.J., Yang H., Xian T., Chen X.J. (2018). Construction of Z-scheme g-C_3_N_4_/CNT/Bi_2_Fe_4_O_9_ composites with improved simulated-sunlight photocatalytic activity for the dye degradation. Micromachines.

[17] Fernando K.A.S., Sahu S.P., Liu Y., Lewis W.K., Guliants E., Jafariyan A., Wang P., Bunker C.E., Sun Y.P. (2015). Carbon quantum dots and applications in photocatalytic energy conversion. ACS Appl. Mater. Interfaces.

[18] Yu H.J., Shi R., Zhao Y.F., Waterhouse G.I.N., Wu L.Z., Tung C.H., Zhang T.R. (2016). Smart utilization of carbon dots in semiconductor photocatalysis. Adv. Mater..

[19] Li H.T., Kang Z.H., Liu Y., Lee S.T. (2012). Carbon nanodots: Synthesis, properties and applications. J. Mater. Chem..

[20] Devi1 P., Thakur A., Bhardwaj S.K., Saini S., Rajput P., Kumar P. (2018). Metal ion sensing and light activated antimicrobial activity of aloevera derived carbon dots. J. Mater. Sci.-Mater. Electron..

[21] Yang Y.N., Xia L., Zhang T., Shi B., Huang L.N., Zhong B., Zhang X.Y., Wang H.T., Zhang J., Wen G.W. (2018). Fe_3_O_4_@LAS/RGO composites with a multiple transmission-absorption mechanism and enhanced electromagnetic wave absorption performance. Chem. Eng. J..

[22] Jiang J.L., He X.X., Du J.F., Pang X.J., Yang H., Wei Z.Q. (2018). In-situ fabrication of graphene-nickel matrix composites. Mater. Lett..

[23] Liang C.P., Niu G., Chen X.F., Zhou Z.G., Yi Z., Ye X., Duan T., Yi Y., Xiao S.Y. (2019). Tunable triple-band graphene refractive index sensor with good angle-polarization tolerance. Opt. Commun..

[24] Cen C.L., Lin H., Huang J., Liang C.P., Chen X.F., Tang Y.J., Yi Z., Ye X., Liu J.W., Yi Y.G. (2018). A tunable plasmonic refractive index sensor with nanoring-strip graphene arrays. Sensors.

[25] Wang X.X., Wu X.X., Chen Y.Z., Bai X.L., Pang Z.Y., Yang H., Qi Y.P., Wen X.L. (2018). Investigation of wide-range refractive index sensor based on asymmetric metal-cladding dielectric waveguide structure. AIP Adv..

[26] Pang Z.Y., Tong H., Wu X.X., Zhu J.K., Wang X.X., Yang H., Qi Y.P. (2018). Theoretical study of multiexposure zeroth-order waveguide mode interference lithography. Opt. Quant. Electron..

[27] Wang X.X., Pang Z.Y., Tong H., Wu X.X., Bai X.L., Yang H., Wen X.L., Qi Y.P. (2019). Theoretical investigation of subwavelength structure fabrication based on multi-exposure surface plasmon interference lithography. Results Phys..

[28] Xia L., Zhang X.Y., Yang Y.N., Zhang J., Zhong B., Zhang T., Wang H.T. (2018). Enhanced electromagnetic wave absorption properties of laminated SiCNW-C_f_/lithium-aluminum-silicate (LAS) composites. J. Alloys Compd..

[29] Sharma S., Mehta S.K., Ibhadon A.O., Kansal S.K. (2019). Fabrication of novel carbon quantum dots modified bismuth oxide (α-Bi_2_O_3_/C-dots): Material properties and catalytic applications. J. Colloid Interface Sci..

[30] Di J., Xia J.X., Ge Y.P., Li H.P., Ji H.Y., Xu H., Zhang Q., Li H.M., Li M.N. (2015). Novel visible-light-driven CQDs/Bi_2_WO_6_ hybrid materials with enhanced photocatalytic activity toward organic pollutants degradation and mechanism insight. Appl. Catal. B-Environ..

[31] Duo F.F., Wang Y.W., Fan C.M., Zhang X.C., Wang Y.F. (2016). Enhanced visible light photocatalytic activity and stability of CQDs/BiOBr composites: The upconversion effect of CQDs. J. Alloy Compd..

[32] Hong Y.Z., Meng Y.D., Zhang G.Y., Yin B.X., Zhao Y., Shi W.D., Li C.S. (2016). Facile fabrication of stable metal-free CQDs/g-C_3_N_4_ heterojunctions with efficiently enhanced visible-light photocatalytic activity. Sep. Purif. Technol..

[33] Zhao X.X., Yang H., Li S.H., Cui Z.M., Zhang C.R. (2018). Synthesis and theoretical study of large-sized Bi_4_Ti_3_O_12_ square nanosheets with high photocatalytic activity. Mater. Res. Bull..

[34] Zhang Y.Z., Chen Z.W., Lu Z.Y. (2018). A facile method for the preparation of colored Bi_4_Ti_3_O_12−x_ nanosheets with enhanced visible-light photocatalytic hydrogen evolution activity. Nanomaterials.

[35] Gao X.M., Dai Y., Zhang Y., Wang Z.H., Fu F. (2017). Preparation and photocatalytic performance of spherical-like Bi_4_Ti_3_O_12_ composite. Chin. J. Inorg. Chem..

[36] Qian K., Jiang Z.F., Shi H., Wei W., Zhu C.Z., Xie J.M. (2016). Constructing mesoporous Bi_4_Ti_3_O_12_ with enhanced visible light photocatalytic activity. Mater. Lett..

[37] Zhao W., Jia Z., Lei E., Wang L.G., Li Z.Y., Dai Y.J. (2013). Photocatalytic degradation efficacy of Bi_4_Ti_3_O_12_ micro-scale platelets over methylene blue under visible light. J. Phys. Chem. Solids.

[38] Hervoches C.H., Lightfoot P. (1999). A variable-temperature powder neutron diffraction study of ferroelectric Bi_4_Ti_3_O_12_. Chem. Mater..

[39] Cummins S.E., Cross L.E. (1968). Electrical and optical properties of ferroelectric Bi_4_Ti_3_O_12_ single crystals. J. Appl. Phys..

[40] Cui Z.M., Yang H., Zhao X.X. (2018). Enhanced photocatalytic performance of g-C_3_N_4_/Bi_4_Ti_3_O_12_ heterojunction. Mater. Sci. Eng. B.

[41] Zhao Y.W., Fan H.Q., Fu K., Ma L.T., Li M.M., Fang J.W. (2016). Intrinsic electric field assisted polymeric graphitic carbon nitride coupled with Bi_4_Ti_3_O_12_/Bi_2_Ti_2_O_7_ heterostructure nanofibers toward enhanced photocatalytic hydrogen evolution. Int. J. Hydrogen Energy.

[42] Hou D.F., Hu X.L., Hu P., Zhang W., Zhang M.F., Huang Y.H. (2013). Bi_4_Ti_3_O_12_ nanofibers–BiOI nanosheets p–n junction: facile synthesis and enhanced visible-light photocatalytic activity. Nanoscale.

[43] Shi B.T., Yin H.Y., Gong J.Y., Nie Q.L. (2017). A novel p-n heterojunction of Ag_2_O/Bi_4_Ti_3_O_12_ nanosheet with exposed (001) facets for enhanced visible-light-driven photocatalytic activity. Mater. Lett..

[44] Liu Y.B., Zhu G.Q., Gao J.Z., Hojamberdiev M., Lu H.B., Zhu R.L., Wei X.M., Liu P. (2016). A novel CeO_2_/Bi_4_Ti_3_O_12_ composite heterojunction structure with an enhanced photocatalytic activity for bisphenol A. J. Alloy Compd..

[45] Zhao W., Wang H.X., Feng X.N., Jiang W.Y., Zhao D., Li J.Y. (2015). Hydrothermal synthesis and photocatalytic activities of Bi_4_Ti_3_O_12_/SrTiO_3_ composite micro-platelets. Mater. Res. Bull..

[46] Zheng C.X., Yang H., Cui Z.M., Zhang H.M., Wang X.X. (2017). A novel Bi_4_Ti_3_O_12_/Ag_3_PO_4_ heterojunction photocatalyst with enhanced photocatalytic performance. Nanoscale Res. Lett..

[47] Liu Y., Zhang M.Y., Li L., Zhang X.T. (2015). In situ ion exchange synthesis of the Bi_4_Ti_3_O_12_/Bi_2_S_3_ heterostructure with enhanced photocatalytic activity. Catal. Commun..

[48] Cao T.P., Li Y.J., Wang C.H., Zhang Z.Y., Zhang M.Y., Shao C.L., Liu Y.C. (2011). Bi_4_Ti_3_O_12_ nanosheets/TiO_2_ submicron fibers heterostructures: in situ fabrication and high visible light photocatalytic activity. J. Mater. Chem..

[49] Wang T., Liu X.Q., Ma C.C., Zhu Z., Liu Y., Liu Z., Wei M.B., Zhao X.X., Dong H.J., Huo P.W. (2018). Bamboo prepared carbon quantum dots (CQDs) for enhancing Bi_3_Ti_4_O_12_ nanosheets photocatalytic activity. J. Alloy Compd..

[50] Dai J.Y., Li J.J., Zhang Q.B., Liao M., Duan T., Yao W.T. (2019). Co_3_S_4_@C@MoS_2_ microstructures fabricated from MOF template as advanced lithium-ion battery anode. Mater. Lett..

[51] Zheng C.X., Yang H. (2018). Assembly of Ag_3_PO_4_ nanoparticles on rose flower-like Bi_2_WO_6_ hierarchical architectures for achieving high photocatalytic performance. J. Mater. Sci.-Mater. Electron..

[52] Eckert J.O., Hung-Houston C.C., Gersten B.L., Lencka M.M., Riman R.E. (1996). Kinetics and mechanisms of hydrothermal synthesis of barium titanate. J. Am. Ceram. Soc..

[53] Konstantinou I.K., Albanis T.A. (2004). TiO_2_-assisted photocatalytic degradation of azo dyes in aqueous solution: kinetic and mechanistic investigations: A review. Appl. Catal. B-Environ..

[54] Zhao X.X., Yang H., Li R.S., Cui Z.M., Liu X.Q. (2019). Synthesis of heterojunction photocatalysts composed of Ag_2_S quantum dots combined with Bi_4_Ti_3_O_12_ nanosheets for the degradation of dyes. Environ. Sci. Pollut. Res. Int..

[55] Di L.J., Yang H., Xian T., Chen X.J. (2018). Facile synthesis and enhanced visible-light photocatalytic activity of novel p-Ag_3_PO_4_/n-BiFeO_3_ heterojunction composites for dye degradation. Nanoscale Res. Lett..

[56] Shi B., Yin H., Gong J., Nie Q. (2017). Ag/AgCl decorated Bi_4_Ti_3_O_12_ nanosheet with highly exposed (001) facets for enhanced photocatalytic degradation of Rhodamine B, Carbamazepine and Tetracycline. Appl. Surf. Sci..

[57] Du C., Li D.H., He Q.Y., Liu J.M., Li W., He G.N., Wang Y.Z. (2016). Design and simple synthesis of composite Bi_12_TiO_20_/Bi_4_Ti_3_O_12_ with a good photocatalytic quantum efficiency and high production of photo-generated hydroxyl radicals. Phys. Chem. Chem. Phys..

[58] Lin H., Ye X., Chen X.F., Zhou Z.G., Yi Z., Niu G., Yi Y.G., Hua Y.T., Hua J.J., Xiao S.Y. (2019). Plasmonic absorption enhancement in graphene circular and elliptical disk arrays. Mater. Res. Express.

[59] Yan Y.X., Yang H., Zhao X.X., Zhang H.M., Jiang J.L. (2018). A hydrothermal route to the synthesis of CaTiO_3_ nanocuboids using P25 as the titanium source. J. Electron. Mater..

[60] Yu B.Y., Kwak S.Y. (2012). Carbon quantum dots embedded with mesoporous hematite nanospheres as efficient visible light-active photocatalysts. J. Mater. Chem..

[61] Wang S.F., Gao H.J., Fang L.M., Wei Y., Li Y.W., Lei L. (2019). Synthesis and characterization of BaAl_2_O_4_ catalyst and its photocatalytic activity towards degradation of methylene blue dye. Z. Phys. Chem..

[62] Mert B.D., Mert M.E., Kardas G., Yazici B. (2012). Experimental and theoretical studies on electrochemical synthesis of poly(3-amino-1,2,4-triazole). Appl. Surf. Sci..

[63] Ganesh R.S., Sharma S.K., Abinnas N., Durgadevi E., Raji P., Ponnusamy S., Muthamizhchelvan C., Hayakawa Y., Kim D.Y. (2017). Fabrication of the flexible nanogenerator from BTO nanopowders on graphene coated PMMA substrates by sol-gel method. Mater. Chem. Phys..

[64] Dias J.A., Oliveira J.A., Renda C.G., Morelli M.R. (2018). Production of nanometric Bi_4_Ti_3_O_12_ powders: From synthesis to optical and dielectric properties. Mater. Res..

[65] Xie R.Y., Zhang L.P., Liu H.C., Xu H., Zhong Y., Sui X.F., Mao Z.P. (2017). Construction of CQDs-Bi_20_TiO_32_/PAN electrospun fiber membranes and their photocatalytic activity for isoproturon degradation under visible light. Mater. Res. Bull..

[66] Xia Y.M., He Z.M., Lu Y.L., Tang B., Sun S.P., Su J.B., Li X.P. (2018). Fabrication and photocatalytic property of magnetic SrTiO_3_/NiFe_2_O_4_ heterojunction nanocomposites. RSC Adv..

[67] Zhu X.L., Wei Z.Q., Zhao W.H., Zhang X.D., Zhang L., Wang X. (2018). Microstructure and electrochemical properties of ZnMn_2_O_4_ nanopowder synthesized using different surfactants. J. Electron. Mater..

[68] Ye Y.C., Yang H., Wang X.X., Feng W.J. (2018). Photocatalytic, Fenton and photo-Fenton degradation of RhB over Z-scheme g-C_3_N_4_/LaFeO_3_ heterojunction photocatalysts. Mater. Sci. Semicond. Proc..

[69] Fattah-alhosseini A. (2016). Passivity of AISI 321 stainless steel in 0.5 M H_2_SO_4_ solution studied by Mott–Schottky analysis in conjunction with the point defect model. Arab. J. Chem..

[70] Cardon F., Gomes W.P. (1978). On the determination of the flat-band potential of a semiconductor in contact with a metal or an electrolyte from the Mott-Schottky plot. J. Phys. D Appl. Phys..

[71] Wang F., Yang H., Zhang H.M., Jiang J.L. (2018). Growth process and enhanced photocatalytic performance of CuBi_2_O_4_ hierarchical microcuboids decorated with AuAg alloy nanoparticles. J. Mater. Sci.-Mater. Electron..

[72] Ye Y.C., Yang H., Zhang H.M., Jiang J.L. (2019). A promising Ag_2_CrO_4_/LaFeO_3_ heterojunction photocatalyst applied to photo-Fenton degradation of RhB. Environ. Technol..

